# Supplementary figures and images for: Supplementary feeding of cattle-yak in the cold season alters rumen microbes, volatile fatty acids, and expression of SGLT1 in the rumen epithelium
Source: PeerJ. 2021 Mar 18;9:e11048. doi: 10.7717/peerj.11048 (PMC7982075; doi:10.7717/peerj.11048)

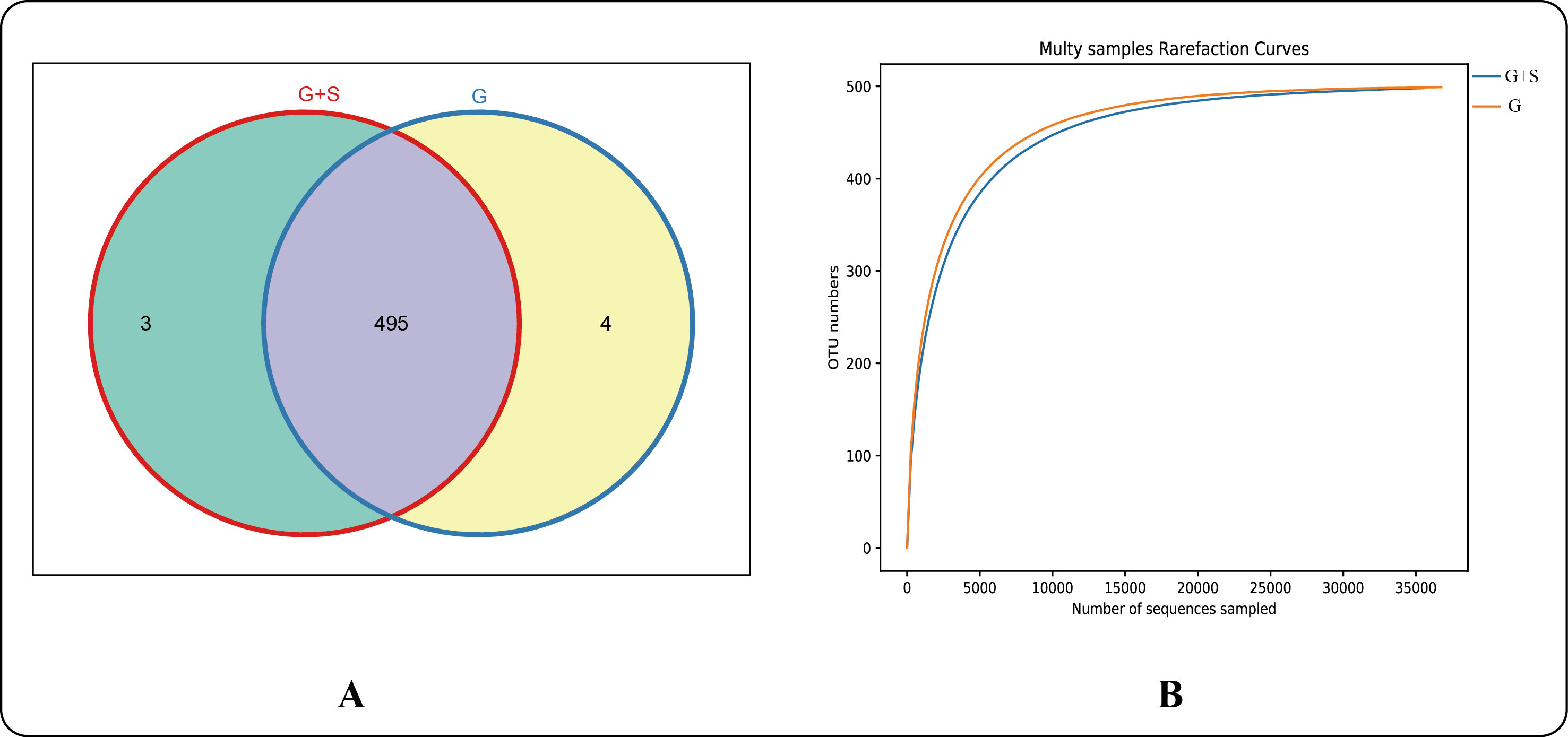

Supplement: Supplemental Information 6 — G: grazing; G+S: grazing + supplementary feeding; OTU: operational taxonomic unit. [file peerj-09-11048-s006.png]
